# Outcomes of Robotic Transabdominal Retromuscular Repair: 3-Year Follow-up

**DOI:** 10.3389/jaws.2024.12907

**Published:** 2024-06-20

**Authors:** Aquiles Garza, Cesar Amaya-Romero, Gabriel Arevalo

**Affiliations:** Department of Surgery, Houston Methodist Willowbrook Hospital, Houston, TX, United States

**Keywords:** ventral hernia, long-term, r-TARUP, incisional hernia, EuraHS-QoL, umbilical hernia, retromuscular

## Abstract

**Background:**

Our study addresses the gap in ventral hernia repair literature, regarding the long-term effectiveness of robotic transabdominal retrorectus umbilical prosthetic repair (r-TARUP) for primary and incisional ventral hernias. This study aimed to report the 3-year recurrence rates and overall patient outcomes including quality of life.

**Method:**

A retrospective review of prospective collected data analyzed 101 elective r-TARUP patients from August 2018 to January 2022. Data collected included demographics, hernia sizes, mesh types, postoperative outcomes and the European Hernia Society Quality of Life questionnaire (EuraHS-QoL) before and after surgery.

**Results:**

The average age of the group of patients was 53, having a mean body mass index (BMI) of 32 kg/m, with 54% incisional and 46% primary hernias, with mean length and width of 4.4 cm and 6.1 cm, utilizing synthetic 58% and bioabsorbable 42% mesh types. The majority were classified as Centers of Disease Control and Prevention (CDC) class I wounds. Postoperative complications included seroma (2%), hematoma (3%), which required surgical intervention, with no significant correlation to mesh type. A strong positive correlation was found between Transversus Abdominis Release (TAR) and increased length of hospital stay (correlation coefficient: 0.731, *p* < 0.001). Preoperative quality of life assessments demonstrated statistically significant improvements when compared to postoperative assessments at 3 years, with a mean (±SD) of 61.61 ± 5.29 vs. 13.84 ± 2.6 (*p* < 0.001). Mean follow up of 34.4 months with no hernia recurrence at 1 year and 3 recurrence at the 2-3 years follow up (3.2%).

**Conclusion:**

The r-TARUP technique has proven to be safe and effective for repairing primary and incisional ventral hernias, with a low recurrence rate during this follow up period with a noticeable improvement in quality of life (QoL).

## Introduction

Minimally invasive transabdominal approach to the retromuscular plane for ventral hernia repair has been a topic of interest in the field of surgical abdominal wall reconstruction. Chowbey et al. [1] and Schroeder [2] initially described this approach using a laparoscopic platform. Chowbey reported an increased amount of dissection resulting in increased operative time; Schroeder reported it to be a technically demanding procedure, and similarly reported increased operative times. The robotic transabdominal retromuscular umbilical prosthetic hernia repair (r-TARUP) described by Dr. Filip Muysoms in 2018 [3] was developed to ameliorate the challenges encountered during the lateral transabdominal laparoscopic approach. Muysom was noted to have a shorter operative time than that of the laparoscopic transabdominal retrorectus technique described in the literature. Using the robotic platform through a single-dock lateral approach facilitates the dissection of the planes, and wrist instruments improve suturing of the ipsilateral posterior rectus sheath, thereby improving the overall operative time. Minimally invasive transabdominal approach to the retromuscular plane for ventral hernia repair has evolved over the years.

The use of robotic transabdominal retrorectus hernia repair has been expanded to include the repair of concomitant rectus diastasis by Cuccurullo et al. [4, 5] with a 1-year follow-up and for more complex abdominal wall pathologies, such as the management of parastomal hernias, first described by Maciel et al. [6]. These studies demonstrated the safe, reproducible, and potential applications of robotic transabdominal wall pathologies including concomitant rectus diastasis and parastomal hernias. However, there is limited information regarding the long-term outcomes of transabdominal retrorectus repair in the treatment of primary and incisional ventral hernias.

This study aims to present the 3-year recurrence rates and identify factors that may predict hernia recurrence. Additionally, we aim to report on the preoperative and postoperative quality of life scores, utilizing a hernia-specific quality of life assessment tool.

## Materials and Methods

### Study Design

In accordance with Institutional Review Board (IRB) approval, a retrospective analysis of prospectively collected data was performed on patients who underwent the robotic transabdominal retrorectus approach from August 2018 to January 2022 at a single institution. The inclusion criterion was the use of r-TARUP for the treatment of primary ventral and incisional hernias in patients aged 18 years and older. Excluded from the study were patients who underwent hybrid robotic abdominal wall repair, as well as those with flank hernias, or parastomal defects. Patients who underwent laparoscopic surgery were excluded from the study. The American Society of Anesthesiologist (ASA) classification of 4 were excluded from the study. The database was reviewed for demographics, risk factors, hernia size, hernia type, mesh type and size, surgical outcomes, length of hospital stay, and return to work. Hernia defect characteristics adhered to the current ventral hernia classification guidelines by the European Hernia Society [7].

Surgical outcomes included Wound Classification according to the Centers for Disease Control and Prevention guidelines: I Clean, II Clean-Contaminated, III Contaminated, IV Dirty [8], Length of Stay, Return to Work, Surgical Site infection (SSI), Surgical Site Occurrence (SSO), and Surgical Site Occurrence requiring Procedural Intervention (SSOPI) [9]. The SSO classification adhered to the VHWG [10], including seroma, wound dehiscence, enterocutaneous fistula, cellulitis, hematoma, and delayed wound healing. We also measured the recurrence rates and administered the validated hernia-specific quality of life questionnaire.

**FIGURE 1:**
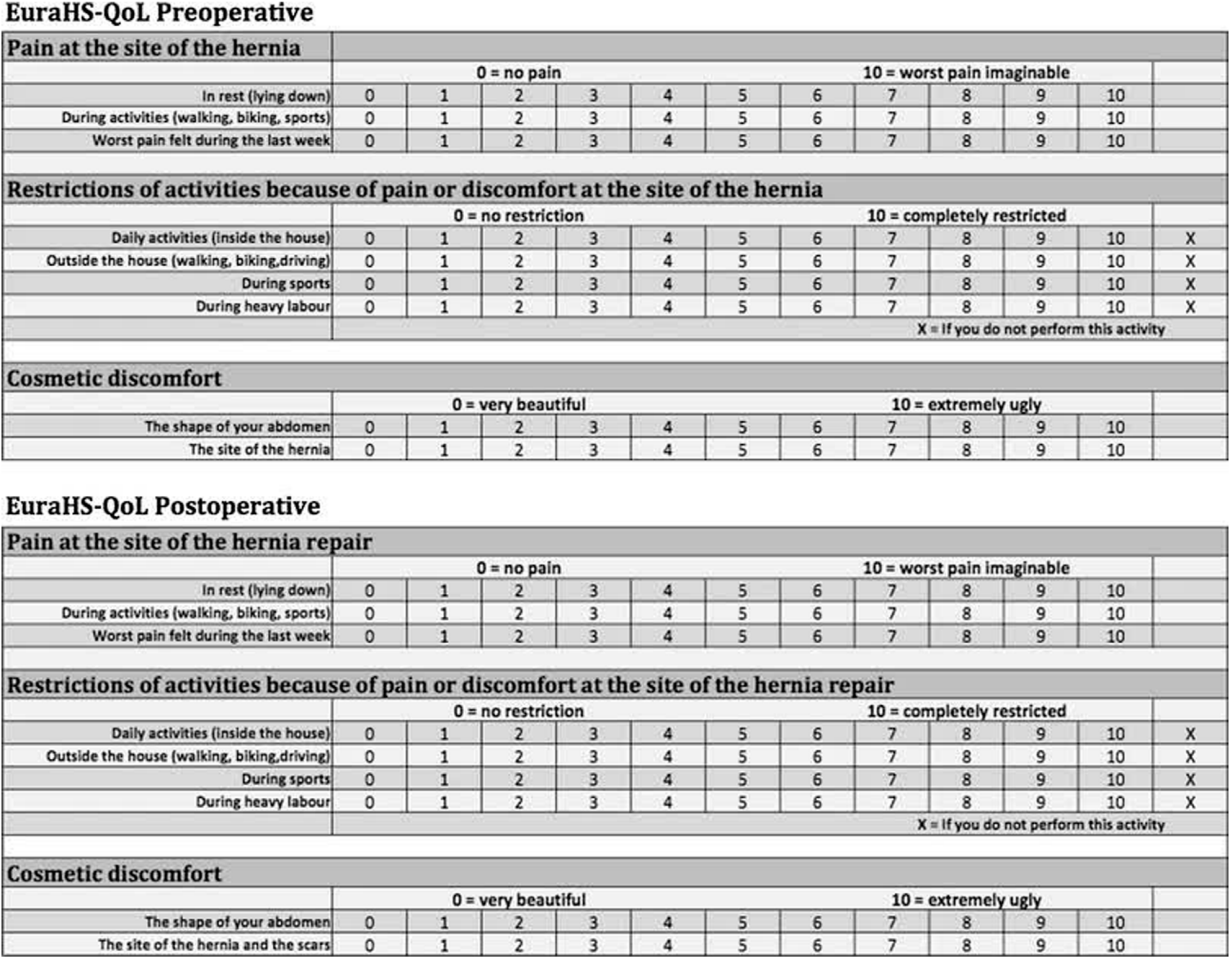
European hernia Society quality of life questionnaire.

Our study utilized the European Hernia Society Quality of Life (EuraHS-QoL) questionnaire, proposed by the European Hernia Society Working Group [11]. Developed with significant contributions from Dr. Filip Muysoms. This specialized instrument focuses on three critical variables: pain, activity limitations, and cosmetic discomfort. It provides a straightforward and comprehensive evaluation of a patient’s wellbeing. Each variable is scored on an 11-point scale, ranging from 0 (no discomfort) to 10 (severe discomfort); with the domain scores summed to produce a total score from 0 to 90. Lower scores indicate a better quality of life, while higher scores suggest a worse quality of life (Figure 1). The EuraHS-QoL’s capability to assess patients before and after surgery, along with its validated effectiveness and user-friendliness, led us to prefer it over other instruments. For instance, the Hernia-Related Quality of Life Survey (HerQLes) [12], although similar, does not effectively capture more subjective aspects of quality of life such as cosmesis and is more cumbersome to complete. Moreover, the Carolinas Comfort Scale (CCS) [13, 14], while detailed, requires answering 23 questions, which can also be cumbersome during phone interviews. Its trademarked status also necessitates a tedious licensing process and restricts publishing in open access journals.

In this study, the handling of missing data for the quality of life questionnaire was guided by a validated method developed by Filip Muysoms [15] [Table 1]. The purpose of these criteria for managing missing values is to address discrepancies that may arise when patients respond to the questionnaire. Such discrepancies can stem from human error, misunderstandings of the questions, or specific responses like “I do not perform this activity” in the domain addressing restrictions of activities.

**TABLE 1:** Validating the EuraHS-QoL for missing data.

Domain	Condition	Action Taken
Pain Domain		
	1 question unanswered	Replace with the mean of the two answered questions
	2 or 3 questions unanswered	Domain score considered missing
Restrictions Domain		
	1 or 2 questions unanswered	Replace missing values with the mean of answered questions
	3 or 4 questions unanswered	Domain score considered missing
Cosmetic Domain		
	1 question unanswered	Replace the missing value with the score from the other question
	Both questions unanswered	Domain score considered missing
Overall Score		
	1 domain score missing	Use the mean of the remaining two domain scores
	2 or more domain scores missing	Overall score considered missing

Note: Muysoms et al. [15].

Follow-up occurred at 2 weeks, 3 months, 12 months, and 2-3 years postoperatively. Quality of life assessments were conducted preoperatively and at 3 months, 12 months and 3 years postoperative. For follow-ups beyond 12 months, a telephone questionnaire was administered at two and 3 years using the standardized Validated Ventral Hernia Repair-Telephone Survey (VHR-TS) [16] [Table 2], along with the EuraHS-QoL questionnaire. In-office visits were scheduled if hernia-related complications were suspected.

**TABLE 2:** Validated ventral hernia repair-telephone survey (VHR-TS).

1. Do you feel that your hernia is back?
2. Has any physician told you that your hernia is back?
3. Do you have a bulge/lump where your hernia used to be?
4. Do you have any painful areas on your abdominal wall?
A positive answer to any of the questions is considered a recurrence until proven otherwise

Note: Novitsky et al. [16].

### Setting

The study was conducted at Willowbrook Methodist Hospital in Houston, Texas, a regional teaching hospital, by two surgeons employing the Intuitive Da Vinci Xi Surgical platform.

### Standardized Work-Up Protocol

All patients received comprehensive information through oral and presurgical documentation. Ventral hernias were meticulously classified following the guidelines set by the European Hernia Society (EHS) [7] and measured using dynamic abdominal ultrasonography (US) or computed tomography (CT) [17]. Following informed consent, each patient with a complex ventral hernia underwent a specialized Enhanced Recovery After Surgery (ERAS) [18–20] protocol tailored to hernia-specific needs. Additionally, patients completed the preoperative EuraHS-QoL questionnaire. We provided active counseling and support to ensure that patients achieved smoking cessation for at least 4 weeks before surgery, achieved optimal glycemic control for diabetic patients, and maintained an optimal mental, physical, and nutritional status.

### Standardized r-TARUP Technique

Our standard lateral approach for the r-TARUP procedure begins with establishing pneumoperitoneum at 12 mmHg using a Veress needle. Three 8 mm trocars are placed laterally along the anterior axillary line.

**FIGURE 2:**
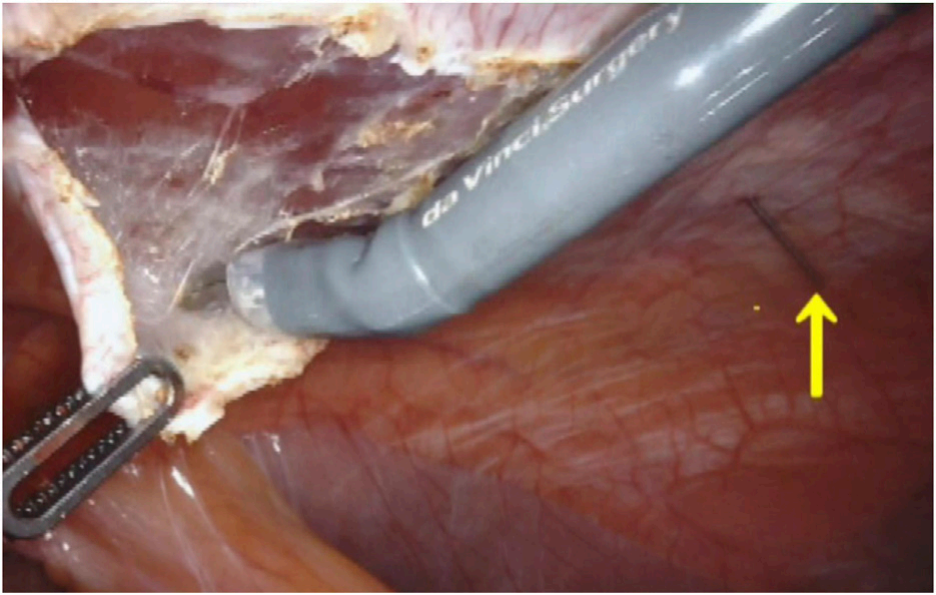
Spinal needle preventing medial or lateral deviation of the incision after identification of the rectus muscle fibers.

The Da Vinci Xi robot is docked from the patient’s right side. Adhesiolysis and hernia content reduction proceed. The ipsilateral PRS is opened at least 5 cm from the hernia’s lateral border. Transabdominal spinal needles, placed by the bedside assistant, help correct the orientation of the PRS longitudinal incision to avoid lateral deviations or medial wandering (Figure 2).

The longitudinal muscle fibers of the left rectus muscle are exposed, and a lateral-to-medial dissection in the retromuscular space is performed until the junction between the anterior and posterior rectus fascia is identified.

**FIGURE 3:**
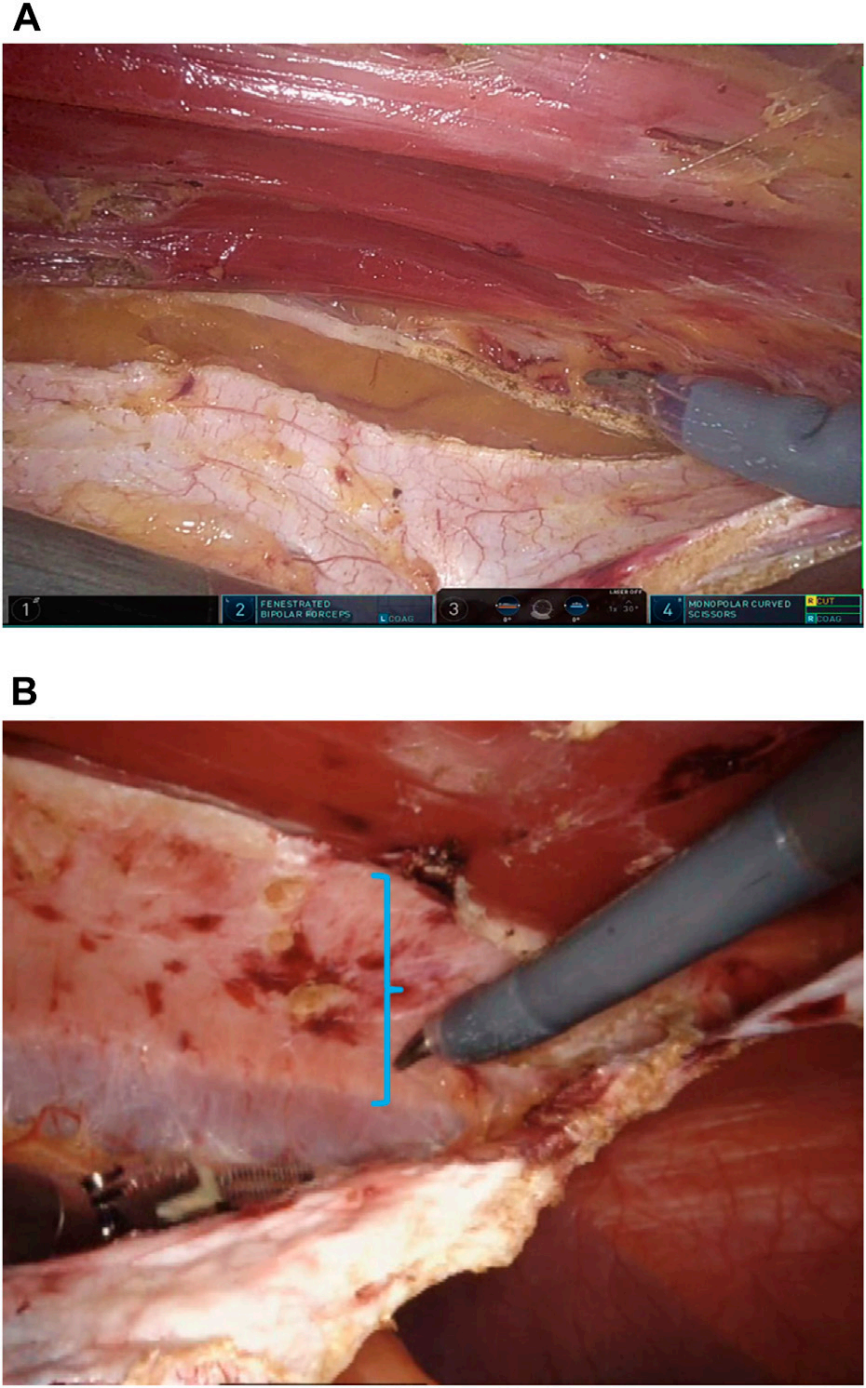
**(A)** Opening of the posterior rectus fascia 0.5–1 cm before its junction with the anterior rectus sheath. **(B)** Diastasis highlighted. Shadowing fibers of the contralateral rectus muscle coming into view.

**FIGURE 4:**
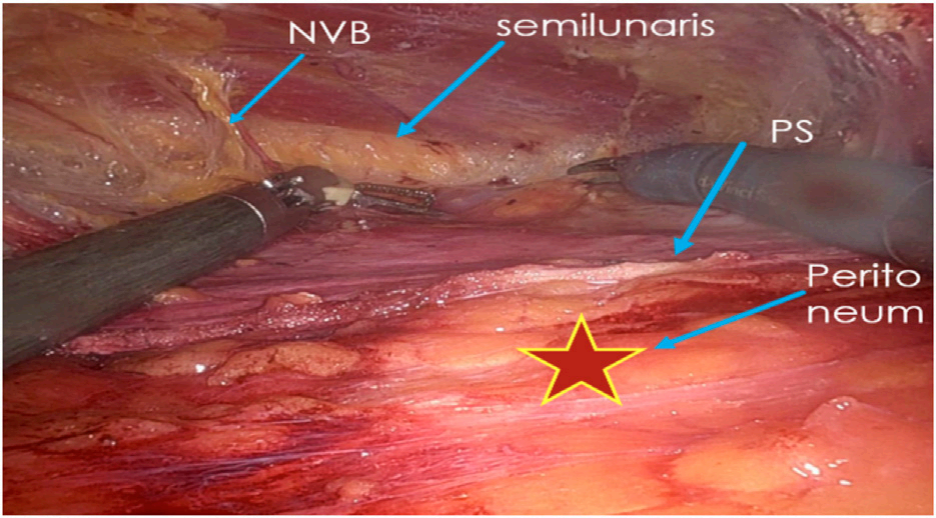
Medial to lateral dissection within the contralateral retrorectus space.

**FIGURE 5:**
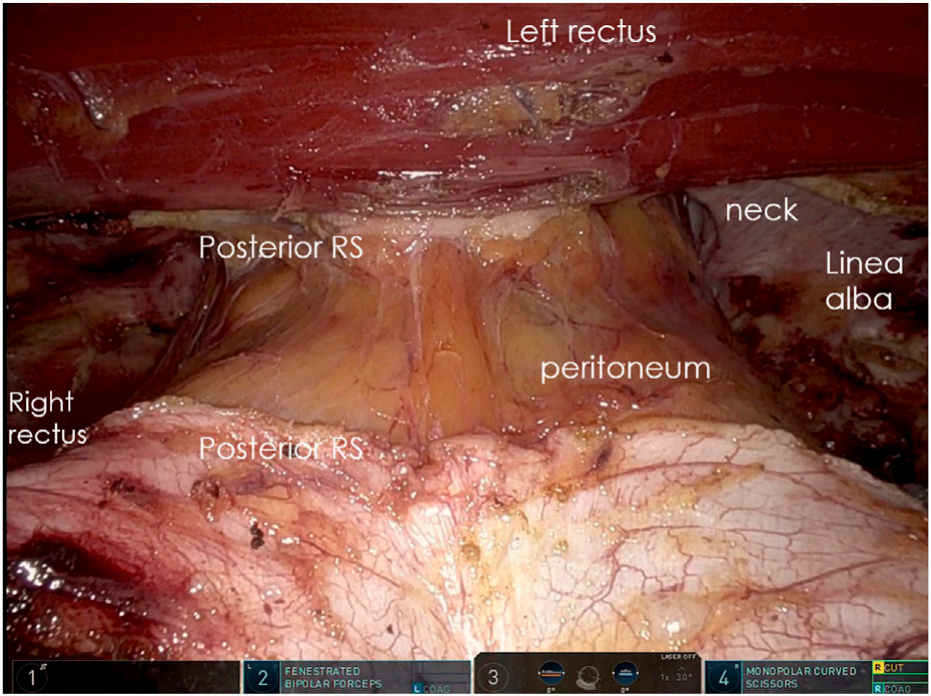
Volcano sign. Bilateral retrorectus space connected medially by the bridging peritoneum.

A crossover maneuver is initiated by incising the medial aspect of the PRS approximately 0.5–1 cm from its junction with the anterior sheath, granting access to the preperitoneal space. During this, the linea alba is kept ventral and the peritoneum dorsal, and any concomitant diastasis is evaluated (Figures 3A, B). The contralateral PRS is then opened, and retrorectus dissection progresses from medial to lateral, identifying the perforating neurovascular bundles and linea semilunaris (Figure 4). Once cranial and caudal dissections adjacent to the hernia defect are completed, the so-called “volcano sign” is achieved (Figure 5), hernia sac and preperitoneal fat reduction proceeds.

If inadequate mesh overlap, increased tension during midline closure, or large peritoneal fenestrations are encountered, unilateral robotic Transversus Abdominis Release (r-TAR) may be safely performed, as described by Novitsky et al. [21].

**FIGURE 6:**
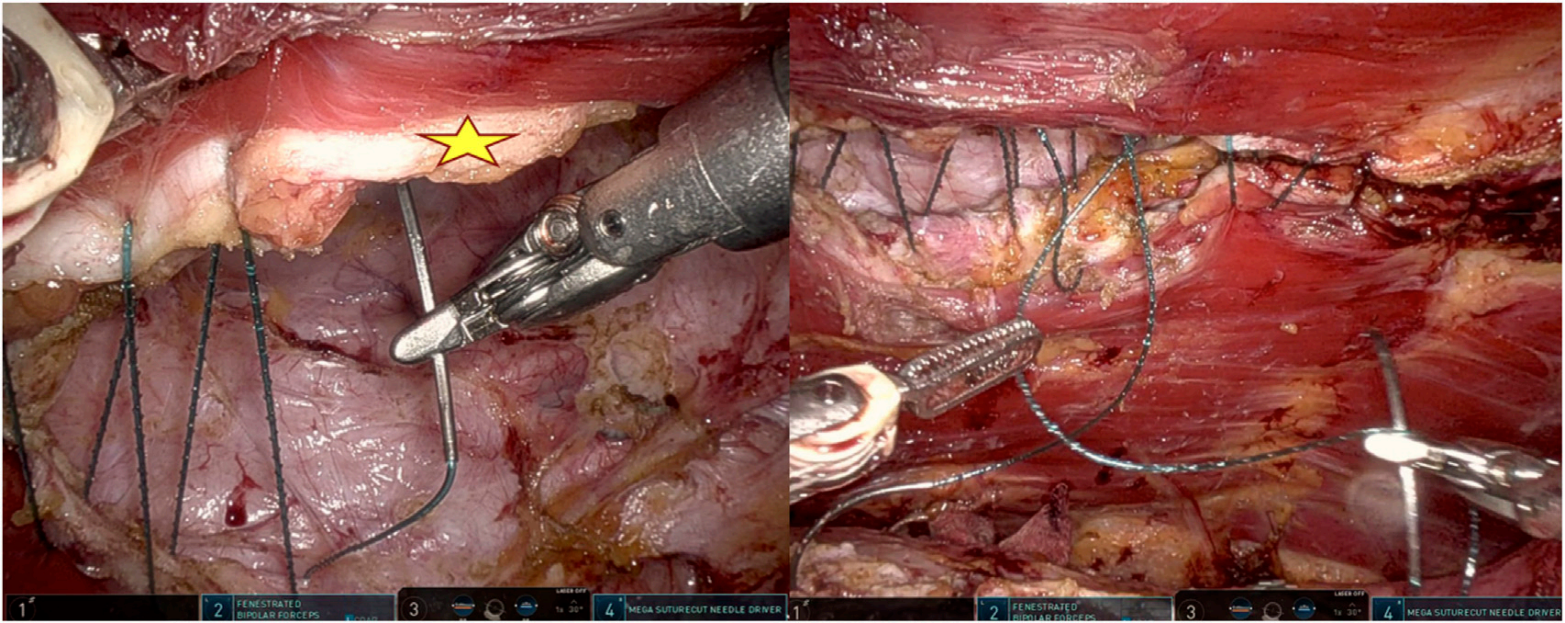
Closure of the hernia defect. Star marks the medial edge of the posterior rectus sheath. For larger defects, closing the cranial and caudal edges first can help to decrease and distribute the tension.

**FIGURE 7:**
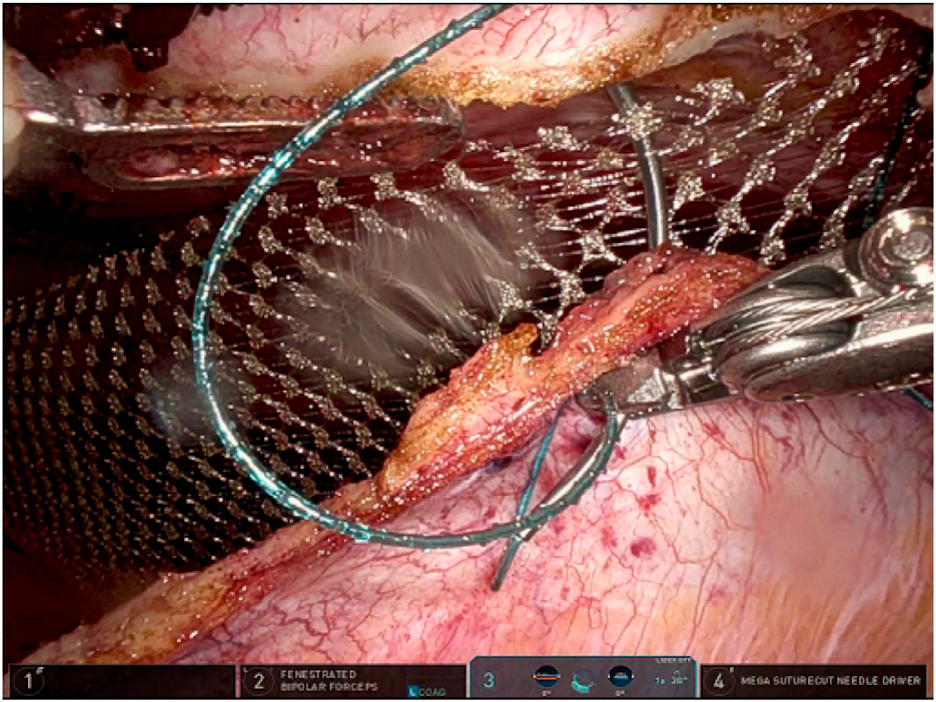
Closure of the ipsilateral posterior rectus sheath with a running absorbable barbed suture incorporating the mesh edge.

The anterior fascial defect is closed with a running 1-0 absorbable barbed suture for synthetic mesh and a 2-0 non-absorbable suture for bioabsorbable mesh (Figure 6). Plication of the hernial pseudo-sac is performed to reduce the risk of seroma formation. For larger hernia sacs, a 15 Blake Jackson-Pratt drain is inserted into the sac to decrease seroma formation. If diastasis was present, inward plication using a horizontal mattress suture is performed to minimize postoperative midline vertical ridges, especially in thin patients. Mesh is inserted within the retromuscular space, typically without fixation. Finally, the ipsilateral posterior rectus sheath is closed with an absorbable 3-0 barbed suture, incorporating the ipsilateral mesh edge into the suture line at the cranial and caudal borders (Figure 7).

### Mesh Selection and Suture Rationale

In our study, the choice of mesh type was strategically guided by clinical scenarios, surgeon preferences, and patient requests. Primary ventral hernias and all clean-contaminated cases were repaired using absorbable mesh, per the surgeon’s preference. We avoided using absorbable sutures with absorbable mesh to prevent suture absorption or fracture during the mesh absorption period and potential rapid hydrolysis before integration. Instead, we used permanent sutures, crucial in the critical post-surgery weeks, to ensure mesh integration and load transfer. Permanent sutures also prevent bridging defects that could cause hernia recurrence if absorbable sutures dissolve prematurely. This hypothesis requires further validation.

For incisional hernias, which demonstrate different outcomes compared to ventral hernias [22], we prioritized optimizing prognosis. Consequently, we selected polypropylene mesh due to its well-documented long-term efficacy in the literature.

### Statistics

In this study, continuous variables were presented as mean ± standard deviation (SD), while categorical variables were expressed as frequency (proportion). Comparative analyses were performed to examine the differences in numerical outcomes, and in categorical outcomes. Specific statistical tests included t-tests for comparisons of means, particularly for analyzing the impact of variables such as age and Body Mass Index (BMI) on surgical outcomes. Chi-square and Fisher’s exact tests were used for comparisons of categorical data, such as for evaluating the association of mesh types with postoperative complications. The Wilcoxon signed-rank test was used to compare paired data, specifically in the analysis of preoperative and postoperative EuraHS-QoL scores at 3 years follow-up. Point-Biserial correlation was applied to assess relationships involving numerical and binary variables, such as examining the association between Transversus Abdominis Release and the length of hospital stay. All tests were two-sided, and a *p*-value < 0.05 was considered to indicate statistical significance.

Statistical analyses were conducted using Python (Version 3.12.0, Wilmington, Delaware) on the Jupyter Notebook, supported by libraries including Pandas, SciPy, and Matplotlib. Microsoft Excel was used for initial data organization and preliminary analysis.

## Results

### Patient Demographics

A total of 101 patients who underwent r-TARUP mean age was 53 years (±13 years). The mean BMI was 32 kg/m2, indicating that the patient group was primarily in the overweight to obese category, surgical site conditions were predominantly “Clean” with 97% of cases, followed by “Clean-Contaminated” cases constituting 3% of the total [Table 3]. Regarding hernia types, 53% of the patients had incisional hernias, while the remaining 4% had primary ventral hernias. The dimensions of the hernia fascial defects had a mean width of 6.1 cm and a mean length of 4.4 cm [Table 4]. In all repairs procedures, defect closure was achieved in all the patients. Hernia repair was reinforced with mesh placement in the sublay space for all patients; 57% of cases utilized synthetic mesh, and 42% employed bioabsorbable mesh, while the latter required permanent suture 0 V-loc for the defect closure.

**TABLE 3:** Patient demographics.

	Patients (*n* = 101)	
Age, years mean ± SD [range]	53 ± 13.3	[28–81]
Gender, n (%)		
Female	43 (42.6)	
Male	58 (57.4)	
BMI, kg/m^2^ mean ± SD [range]	32.10 ± 5.6	[20.3–47]
Comorbities, *n*(%)		
Diabetes	9 (8.91)	
COPD	6 (5.94)	
Immunosupression	8 (7.92)	
Morbid Obesity	9 (8.91)	
Smoker, *n*(%)	11 (10.89)	
ASA Classification, *n* (%)		
Class I	13 (12.87)	
Class II	77 (76.23)	
Class III	11 (10.89)	
Wound class, *n*(%)		
Clean	98 (97.1)	
Clean contaminated	3 (2.9)	
Contaminated	0 (0)	

Morbid obesity BMI ≥40 kg/m^2^.

BMI, body mass index; ASA, american society of anesthesiologists; COPD, chronic obstructive pulmonary disease.

**TABLE 4:** Hernia and mesh characteristics.

	Patients (*n* = 101)
Hernia size, cm	
Length	4.4 ± 1.5
Width	6.1 ± 1.1
Hernia type, *n*(%)	
Incisional	54 (53.4)
Primary	47 (46.5)
Mesh type, *n*(%)	
Synthetic	58 (57.4)
Bioabsorbable	43 (42.6)
Mesh size, cm^2^	105.05 ± 44.92

Mean ± Standard Deviation.

In patients who required unilateral Transversus Abdominis Release (TAR), a strong positive correlation was observed with an increased length of hospital stay (correlation coefficient: 0.731, *p* < 0.001) [Table 5].

**TABLE 5:** Patient surgical outcomes.

	ASPO	2 WPO	3 MPO	12 MPO	2-3 YPO
	*n* = 101	*n* = 101	*n* = 101	*n* = 101	*n* = 92
Surgical Site Occurrence, *n*(%)					
Seroma	–	–	2 (1.9)	–	–
Hematoma	–	3 (2.9)	–	–	–
Delayed wound clossure	–	1 (1.4)	–	–	–
Surgical Site Occurrence Requiring Procedural Intervention, *n*(%)	–	3 (2.9)	2 (1.9)	–	–
Surgical Site Infection, *n*(%)	–	1 (0.9)	–	–	–
Recurrence, *n*(%)	–	–	–	–	3 (3.2)
TAR, *n*(%)	19 (18.8)	–	–	–	–

ASPO, after surgery postoperative; WPO, weeks postopertive; MPO, months postoperative; YPO, years postopertive.

Surgical Wound class CDC guidelines.

TAR, transversus abdominis release.

### Post-Operative Complications

Postoperative complications included symptomatic seroma (2%) (2/101) in the subcutaneous space at 1–3 months postoperative and hematoma (3%) (3/101) in the retromuscular space at 2 weeks postoperatively (Table 6). One patient had delayed wound closure due to skin burn at the umbilicus. There were no statistically significant differences in complications related to mesh type, with *p*-values of 0.611 for seroma and 0.416 for hematoma.

**TABLE 6:** Hospital stay & return to work outcomes.

		*r*
Length of stay, days mean ± SD		
No TAR	<0.1±0.23	0.731*
w/TAR	2.3 ± 0.47	
Return to work, days mean ± SD	6.2 ± 1.2

TAR, transversus abdominis release.

* *p*-value significance, *p* < 0.001.

Point-biserial correlation coefficient.

Surgical site occurrence requiring procedural intervention was 5% (5/101), of which two patients required drainage of seroma, one evacuation of hematoma from the retromuscular space.

The mean follow up of 34.4 months (range 4–42 months), with no hernia recurrence within the first year follow up. Three hernia recurrences were reported at 3-year follow-up. Nine patients were considered lost to follow up beyond the 12 months follow up period, after three phone call attempts and one email, representing a 91.09% retention rate.

Hernia recurrences were repaired robotically, with a preperitoneal repair for an epigastric defect in a patient with diastasis extending to the xiphoid process. The other two recurrences were repaired using the intra-abdominal preperitoneal underlay mesh (IPUM) technique.These two recurrences were related to decreased mesh overlap at the opening of the posterior rectus sheath flap. Two recurrences occurred with synthetic polypropylene mesh and one with bioabsorbable mesh, the latter in the epigastrium of a patient with concomitant diastasis that was not addressed in the initial surgery. Computed tomography imaging showed the recurrence at 2 years and 6 months postoperatively (Supplementary Figure).

### Patient-Reported Quality of Life

**FIGURE 8:**
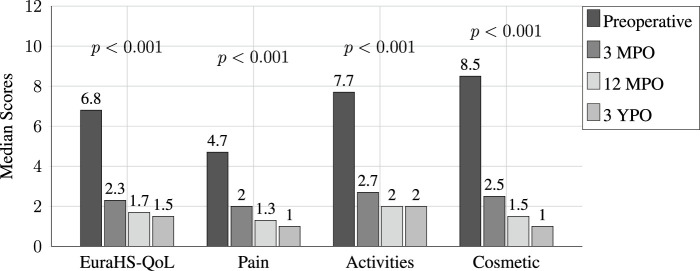
Preoperative vs. Postoperative overall.

The European Hernia Society Quality of Life (EuraHS-QoL) scores used in our study exhibited substantial postoperative improvements. Assessments were conducted preoperatively and at 3 months, 12 months, 2 years, and 3 years postoperatively. The overall mean score decreased significantly at 3 months (61.61 ± 5.29 vs. 21.25 ± 4.75, *p* < 0.001) [Table 7]. Individual domain median scores also improved significantly at 3 months, with pain scores decreasing from 4.7 to 2.1, restriction of activities scores from 7.7 to 2.7, and cosmetic discomfort scores from 8.6 to 2.5. These changes were statistically significant (Wilcoxon signed-rank test), demonstrating the positive impact of surgery on the patients’ quality of life. The decrease in cosmetic scores was particularly significant, indicating greater improvement in this domain compared to pain and restriction of activities at all postoperative time points [Table 8] (Figure 8).

**TABLE 7:** Overall scores EuraHS-QoL questionnaire.

Total overall scores		
Preoperative, *n* = 101		
Mean ± SD	61,61	5,29
Range	48,24	72,93
Median (P25-P75)	61,99	(58.78–65.38)
3 Months Posoperative, *n* = 101		
Mean ± SD	21,25	4.75*
Range	12	31,03
Median (P25-P75)	21,42	(18.09–24.01)
12 Months Posoperative, *n* = 101		
Mean ± SD	16,32	3.33*
Range	7,04	24
Median (P25-P75)	16,81	(14.08–24.01)
3 Years Posoperative, n = 92		
Mean ± SD	13,84	2.6*
Range	5,02	20
Median (P25-P75)	14,19	(12.08–16.01)

*significant results compared to preop, *p* < 0.001.

Wilcoxon signed rank test for *p*-significance.

**TABLE 8:** Domain scores EuraHS-QoL questionnaire.

	*n* = 101	*n* = 101	*n* = 101	*n* = 92
	Preoperative	3 MPO	12 MPO	3 YPO
EuraHS-QoL, mean ± SD [median]	6.8 (0.5) [6.8]	2.3 (0.5) [2.3]*	1.8 (1.2) [1.7]*	1.5 (0.2) [1.5]*
Pain	4.7 (0.6) [4.7]	2.1 (0.6) [2]*	1.2 (0.9) [1.3]*	1.2 (0.34) [1]*
Activities	7.5 (1.7) [7.7]	2.7 (1.4) [2.7]*	2.3 (1.4) [2]*	2.03 (0.6) [2]*
Cosmetic	8.6 (0.8) [8.5]	2.03 (0.9) [2.5]*	1.6 (0.9) [1.5]*	1.1 (0.5) [1]*

MPO, months postopertive; YPO, years postopertive; EuraHS-QoL, European Hernia Society quality-of-life.

*significant results compared to preop, *p* < 0.001.

Wilcoxon signed rank test for *p*-significance.

The use of corrected -values in this longitudinal study accounted for multiple comparisons, thus averting the risk of false positives. The substantial “Statistic” values derived from the repeated-measures ANOVA (F-statistic = 23980.73, *p* < 0.001) and pairwise t-tests with Bonferroni correction confirmed that there were statistically significant changes in QoL scores from preoperative to 3 years postoperatively across the four time points. All comparisons remained highly significant (*p* < 0.001) even after adjustment for multiple comparisons, indicating that enhancements in QoL were consistently significant at each pairwise comparison of time points.

## Discussion

Our findings reveal a compelling narrative about the advantages of r-TARUP, showing a notably low recurrence rate of 2.97%, with no statistical significance based on the type of mesh used. Quality of life improvements were particularly notable in the immediate postoperative period and were sustained over the 3-year follow-up period.

This study provides a comprehensive analysis of the r-TARUP hernia repair technique and reflects its efficacy and implications for hernia repair. The technique’s ability to facilitate closure of hernia defects, as reported in our results, with mean defect width measurement of 6.1 [Table 4], highlights its effectiveness in addressing small to moderate size hernias, although larger hernias W3 ( > 10 cm) can be address with adjuvant unilateral Transversus Abdominis muscle Release (TAR), further enhancing its versatility.

In cases where there was tension in closing the hernia defect or the posterior rectus sheath flap did not provide sufficient overlap for the mesh ipsilaterally, our approach included a TAR procedure as an adjunct to retrorectus release. As described by Novitsky et al. [21, 23, 24], this technique involves opening the posterior lamella of the internal oblique muscle, medial to the linea semilunaris. This process exposes the transversalis muscle, allowing it to be divided and released from its fascia, thus providing additional medialization of the anterior fascia and rectus muscle.

A key feature of the r-TARUP technique is its ability to facilitate mesh placement in a well-vascularized retrorectus space. This strategic placement is significant because it avoids mesh placement within the abdominal cavity, thereby potentially reducing the complications associated with intraperitoneal mesh placement. A disadvantage of r-TARUP repair is the ipsilateral opening of the posterior rectus sheath to access the retrorectus space. Improper closure can lead to intraparietal hernias. Therefore, it is crucial to ensure that the posterior rectus sheath is properly closed at the end of the procedure with careful checks for rent in the peritoneum or sheath. Additionally, improper lateral opening of the sheath without precise ultrasound guidance or anatomical delineation increases the risk of neurovascular bundle injury [25]. Such injury could lead to rectus muscle atrophy and bulging.

In our study, we found that all hernia defects were successfully closed by reconstructing the linea alba, which is crucial for ensuring the integrity of abdominal wall repair. The use of both synthetic and bioabsorbable meshes in our study aligns with the current trends in hernia repair, and offers valuable insights into the effectiveness of different materials.For the bioabsorbable subset of patients, an extended follow-up period of 5 years will be essential to provide comprehensive data on their durability, recurrence rates [26].

Trials in hernia repair have consistently reported improvements in quality of life following minimally invasive techniques for abdominal wall hernia repair [27]. Our study aligns with these findings. In particular, we emphasize the role of hernia-specific questionnaires [13, 28], such as EuraHS-QoL, in accurately capturing patient outcomes. Using this specific assessment in our study provides a deeper and more precise understanding of patients’ before and after surgical experiences. Although other QoL assessments are available, the EuraHS-QoL has been shown to be user-friendly and highly correlated with the CCS, while offering a more detailed and precise evaluation of quality of life [29]. HerQLes, another QoL scale, emphasizes abdominal wall functionality—a pivotal aspect in evaluating functional outcomes related to abdominal wall movement that we may have overlooked by using the EuraHS-QoL scale [12].

One advantage for the EuraHS-Qol is its ability to be validated during inevitable circumstances such as inability to understand the questions making it a precise tool that avoids biases.

Significant improvements were noted from preoperative to 3 years postoperative, with the most substantial improvements observed in the 3 months postoperative period for pain, activity limitations, and aesthetic concerns (Table 8) (Figure 8).

A noteworthy finding of our study was the correlation between the use of posterior component separation TAR and the duration of hospital stay [Table 6]. Patients who did not require the posterior component separation TAR procedure had shorter hospital stays and fewer post-surgery restrictions, highlighting the potential benefits of less invasive techniques for enhancing patient recovery.

The increased hospital stay was due to the surgeon’s preference for careful monitoring of several critical recovery factors. Beyond drain monitoring, the overnight stay allowed for observation of pain, monitoring and adherence to established enhanced recovery protocols for diet and early ambulation which are crucial to patient outcomes. These ERAS principles have been previously described by Fayezizadeh et al. [30] and in recent publications by Marckmann et al. [31].

The complication rates reported with other robotic retromuscular repairs, such as r-TAR and r-eTEP, are significantly low (9%) [32–35]. Postoperative complications in our study occurred at an equal low-frequency, with a seroma rate of 2%, hematoma rate of 3%, and surgical site infection rate of 1%. The literature notes a lack of differentiation between seroma rates within the subcutaneous tissue or retromuscular space. In our study, seromas requiring procedural intervention with a clinical duration greater than 1 month occurred in the subcutaneous space. This rate has decreased since the installation of a tunneled 15 Blake JP drain for large hernial sacs.

Hematomas requiring procedural intervention were located in the retrorectus space and were effectively managed using a laparoscopic approach in two patients, without requiring mesh removal or debridement. The other patient required open hematoma evacuation at the epigastrium and debridement of a small segment of the free-floating mesh. Jackson-Pratt (JP) drain catheters were placed during these interventions. It is important to note that the drains are not routinely used in the TARUP procedure, except in cases where a Transversus Abdominis Release (TAR) procedure is performed. In this subset of patients, none of the JP drains resulted in related complications and the drains were typically removed between postoperative days 7 and 10. This outcome highlights the selective and effective use of JP drains in specific cases within our surgical approach without introducing additional complications.

CDC Class II and III during the robotic incisional hernia repair has been reported to affect the outcomes [36]. In the series, wound contamination occurred in 2.9% of the cases, absorbable mesh was used, surgical site infections occurred in 1%, and the reported surgical site infections did not differ between the clean and contaminated cases. The benefits of minimally invasive repair and inset of wound infection complications are estimated 1.0% [37]. In our study, the average BMI was 32.1 kg/m2 which is quite normal today’s patient population in our geographic area, with an obesity rate of 36.1% [38]. In addition, it was not a predictor of wound infections in our study. The benefits of decreasing wound infection in obese patients by utilizing minimally invasive surgery for hernia repair were evident in our robotic approach, although other patient comorbidities were maximized preoperatively as part of our ERAS pathway, including optimization of diabetes and smoking cessation.

Regarding the subset of patients who underwent absorbable mesh implantation, we believe in the mesh’s ability to integrate with host tissue, supporting fibroblast infiltration and collagen deposition to restore tissue strength [39]. However, longer-term follow-up extending to 5 years or more is crucial to provide more definitive data on the longevity of retromuscular repairs with bioabsorbable mesh (P4HB) and the incidence of late recurrence.

Our study’s 3-year follow-up demonstrated a low recurrence rate of 2.97%, comparable to other MIS retromuscular repairs described by Aliseda et al. [40] We noted that hernia recurrence showed no significant dependence on mesh type. Instead, recurrence rates were related to surgical technique rather than mesh selection. A higher incidence of recurrence was observed in the synthetic mesh group due to decreased mesh overlap.

The decreased mesh overlap at the ipsilateral opening of the posterior rectus sheath is primarily caused by medial wandering during the opening of the PRS. Notably, hernia recurrence in the absorbable mesh group was identified in the epigastrium, particularly at sites of rectus diastasis not fully addressed up to the xiphoid process. To mitigate these issues, our current practice includes the transabdominal placement of spinal needles. This technique helps prevent medial deviation when opening the posterior rectus sheath and ensures complete reconstruction of the linea alba, especially in cases of diastasis.

The r-TARUP technique serves as a robust platform for more complex robotic hernia repair procedures. Its utility extends to techniques such as robotic Extended Totally Extraperitoneal repair (eTEP) and robotic Transversus Abdominis Release (r-TAR), making it a pivotal development in hernia treatment and during the robotic learning curve.

To optimize the application of the r-TARUP technique, it is imperative to understand the abdominal wall anatomy, ensure proper mesh overlap, and address concomitant diastasis to achieve reproducible outcomes. Looking ahead, we advocate for further research on absorbable mesh.

## Strengths and Limitations

### Strengths

The strengths of our study include the 3-year outcomes for the r-TARUP technique, which expand the body of literature on long-term outcomes for ventral hernia repair. Moreover, by incorporating Quality of Life assessments using the EuraHS-QoL scores, we provided a more comprehensive evaluation of patient outcomes. This highlights the positive long-term effects of the r-TARUP technique on patient wellbeing over a 3-year follow-up period.

### Limitations

Limitations of our study include those inherent to a single-institution retrospective study. The study was conducted by two surgeons, which may limit the generalizability of the results to broader populations. Additionally, the relatively small sample size constraints our ability to perform subgroup analyses. However, despite the small sample size, our study has greater power than many existing studies in the r-TARUP literature, for which are limited.

## Conclusion

Our study confirms the safety, efficacy, and enduring success of the r-TARUP technique in treating primary and incisional ventral hernias. The main finding at the 3 years follow up was a low recurrence rate, minimal postoperative complications, and a noticeable improvement in quality of life.

## Data Availability

The original contributions presented in the study are included in the article/Supplementary material, further inquiries can be directed to the corresponding author.
