# Supplementary material for: Outcomes of Robotic Transabdominal Retromuscular Repair: 3-Year Follow-up
Source: J Abdom Wall Surg. 2024 Jun 20;3:12907. doi: 10.3389/jaws.2024.12907 (PMC11222322; doi:10.3389/jaws.2024.12907)
Supplement: Supplementary file 1 [file DataSheet1.docx]

**Supplementary** **figures**.


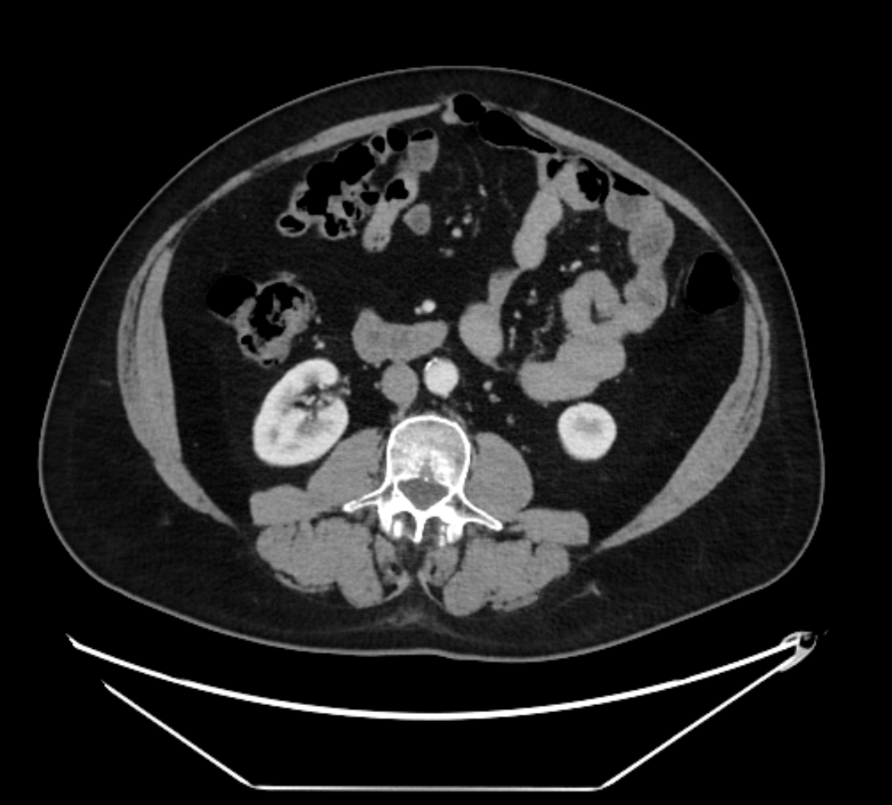


**(Sup. 1.) Recurrence at the diastasis, cranial to the umbilical  hernia repair.**


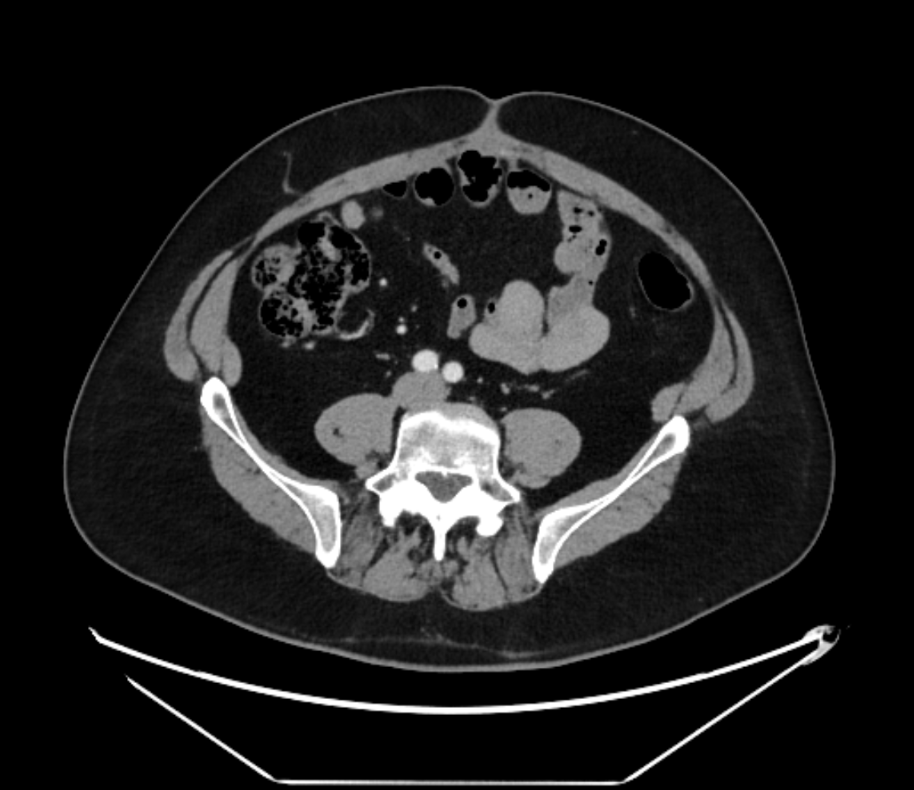


**(Sup. 1.2.) S/p r-TARUP repair, notice the “single motor plate” of the rectus muscle.**
